# Medical cannabis, CBD wellness products and public awareness of evolving regulations in the United Kingdom

**DOI:** 10.1186/s42238-022-00165-6

**Published:** 2022-10-29

**Authors:** Simon Erridge, Ross Coomber, Mikael H Sodergren

**Affiliations:** 1 Department of Surgery & Cancer, Academic Surgical Unit, Imperial College London, 10th Floor QEQM, South Wharf Road, London, W2 1NY UK; 2 Sapphire Medical Clinics, London, UK; 3 St Georges NHS Healthcare Trust, London, UK

**Keywords:** Cannabidiol, Cannabis, Cannabinoids, Medical cannabis, Social stigma

## Abstract

**Background:**

In the UK, legislation and regulations governing medical cannabis and over the counter cannabidiol (CBD) wellness products have rapidly evolved since 2018. This study aimed to assess the public awareness of the availability, regulations, and barriers to access medical cannabis and over the counter CBD wellness products.

**Methods:**

A cross-sectional survey study was performed through YouGov® using quota sampling methodology between March 22nd and March 31st 2021. Responses were matched and statistically weighted to UK adult population demographics, including those without internet access, and analysed according to percentage of respondents. Statistical significance was defined by *p*-value < 0.050.

**Results:**

Ten thousand six hundred eighty-four participants completed the survey. 5,494 (51.4%) respondents believed that medical cannabis is legal in the UK. 684 (6.4%) participants consumed CBD for wellness reasons, 286 (2.7%) were prescribed CBD for a medical reason and 222 (2.1%) consumed CBD for another reason. 10,076 (94.3%) respondents were unaware of April 2021 regulations meaning that all over the counter CBD wellness products in the UK must conform to European Novel Foods Regulations. The most frequently reported main barriers to accessing medical cannabis were its association with recreational cannabis (*n* = 2,686; 25.1%), being unsure if it was legal (*n* = 2,276; 21.3%) and being unsure what medical conditions its can be used for (*n* = 1,863; 17.4%).

**Conclusion:**

A large proportion of respondents are unaware of the legislation and regulations surrounding medical cannabis and over the counter CBD wellness products. Lack of knowledge may present a barrier to safe access to either product.

**Supplementary Information:**

The online version contains supplementary material available at 10.1186/s42238-022-00165-6.

## Introduction

In November 2018, the United Kingdom changed the scheduling restrictions of cannabis-based medicinal products (CBMPs), commonly known as medical cannabis in other jurisdictions (Dowden 2021; Case 2020). This change in scheduling recognised the potential therapeutic properties of medical cannabis products and their constituent active pharmaceutical ingredients, most notably the phytocannabinoids cannabidiol (CBD) and tetrahydrocannabinol (THC) (Dowden 2021; Freeman et al. 2019). Whilst this change facilitated a route to access medical cannabis through appropriate specialists, it has failed to become widely available through the UK’s single-payer healthcare system, the National Health Service (Dowden 2021). The barriers to access are likely multifactorial and consist of stigma, a paucity of high-quality randomised controlled trials, and a lack of awareness and education amongst healthcare professionals and patients (Alexander 2020). With respect to medical cannabis there are inherent challenges in addressing the shortcomings of previously published research (Banerjee et al. 2022). However, the research community is rapidly developing the evidence base across a multitude of conditions for where medical cannabis may be appropriate on a population basis (Erridge et al. 2021; Kawka et al. 2021; Wang et al. 2021; Busse et al. 2021). The challenges in ensuring appropriate awareness and education, however, are likely to have been exacerbated by the speed at which the policy developed during 2018, following a sustained period of unchanged regulations surrounding cannabis (Monaghan et al. 2021). A previous UK survey in 2020 found that only 54% of those sampled knew CBMPs were able to be prescribed by specialists (Hill 2020). However, this survey study was not peer-reviewed and did not report its sampling methodology, so the exact extent to which the UK population is aware of this legislative change is unclear.

In addition to developing policy on medical cannabis, the UK has also recently amended its regulations on CBD oil or other CBD-containing wellness products available without prescription, otherwise known as being available ‘over the counter’. These have been gaining popularity due to the potentiation of beliefs around the therapeutic properties of CBD, which in some cases have conflated the outcomes of research studies with unsubstantiated claims (Pacchetti et al. 2020; Tallon 2020). The UK market for over the counter CBD wellness products is estimated to reach $135 million USD by 2025 (Tallon 2020). On introduction to the market these products were not subject to any specific regulatory challenges. An analysis performed on a selection of over the counter CBD oils by the Centre for Medicinal Cannabis subsequently found that 62% of these oils had CBD concentrations that were more than 10% different to the advertised concentration ﻿(Liebling et al. 2020). Moreover, 55% contained detectable levels of other cannabinoids, such as THC, which are controlled substances (Liebling et al. 2020). Similar findings have also been demonstrated in other countries such as the United States and the Netherlands (Bonn-Miller et al. 2017; Hazekamp 2018). In 2020, the Food Standards Agency introduced guidance that all CBD extracts, utilised either in wellness or food products, be considered as novel foods according to the European Novel Food Regulation (Tallon 2020). This guidance came into force from 1st April 2021, meaning that each over the counter CBD product would require pre-market authorisation to ensure consumer safety (McGregor et al. 2020; Tallon 2020). Due to their status as a novel food, they are also not able to make any claims as to their potential therapeutic value (McGregor et al. 2020). Similar to CBMPs, it is unclear as to whether the public are aware of over the counter CBD wellness products and their changing regulations. Therefore, the aims of this study were to assess what is the public awareness of the availability, regulations, and barriers to access medical cannabis and over the counter CBD wellness products utilising a population-level survey.

## Methods

### Study design

A cross-sectional survey analysis was performed of UK residents between March 22nd and March 31st 2021.

### Study overview

A public-opinion survey study was administered utilising YouGov® (London, United Kingdom) public research panels. Data was collected via *Active Sampling *methodology in accordance with conventional YouGov® polling methodology, whereby participants are invited to partake in interview questions (YouGov 2021; Twyman 2008). Utilising this methodology, in addition to post-hoc weighting, YouGov® ensures that participants are proportionally representative of the UK population according to age, gender, social class, region, education and recent political voting affiliations through utilising quota sampling of a population matched sample (YouGov 2021; Twyman 2008). It has subsequently been proven to be accurate in predicting UK public opinion across politico-social spheres (YouGov 2021; Twyman 2008). YouGov® is a member of the British Polling Council, registered with the Information Commissioner, and a member of European Society for Opinion and Marketing Research (ESOMAR). The research was considered against local ethics approval procedures, and it was determined that this was a policy evaluation and further approval was therefore not required. Participants provided YouGov® with pre-existing consent and as no personal identifying data was provided to the authors, further consent was not required.

### Data collection

There are no previously validated questionnaires to assess knowledge about CBMPs and over the counter CBD products. Therefore, the authors developed the questionnaire via a consensus approach in accordance with the aims of the study and subsequently asked the questions detailed in supplementary Table 1. Pilot testing was performed by the Sapphire Medical Clinics Patient and Public Involvement Group (*n*= 7) to assess content validity and feasibility, with subsequent changes made to the questionnaire based on their recommendations. Questions were asked in series, with simple branching logic applied to questions. In addition, demographic data on each participant was collected, including gender, age, geographic region, working status, marital status, number of children in household, identification, gross household and personal income and house tenure. Social grade of each respondent was determined according to the National Readership Survey (NRS) social grade classifications (Meier & Moy 1999). The responses were statistically weighted by YouGov® according to UK adult population demographics, including people without internet access (YouGov 2021).

### Statistical analysis

Data was analysed using Crunch.IO (Yougov®, London, United Kingdom), embedded within Yougov’s® survey platform. Weighted categorial data including demographic details and questionnaire responses are presented as total number (N) and percentage (%). Differences in responses according to participant demographics were analysed using *Chi-squared* tests. Statistical significance was defined by a *p*-value < 0.050.

## Results

In total 10,684 participants completed the survey within the 10-day period it was open for responses. The weighted population included 5,502 (51.5%) female participants. There were 6,090 (57.0%) participants who were upper or middle class (ABC1) and 4,595 (43.0%) who were working class (C2DE). Participants were from across Great Britain, including England (*n* = 9,231; 86,4%), Scotland (*n* = 930; 8.7%), and Wales (*n* = 524; 4.9%). The full demographic details of participants are detailed in Table 1.

**Table 1:** Demographic data of survey participants

Demographics	n	%
Gender		
Male	5182	48.5%
Female	5502	51.5%
Age		
18–24	1186	11.1%
25–34	1680	15.7%
35–44	1928	18.0%
45–54	1607	15.0%
55+	4282	40.1%
Social Grade		
ABC1	6090	57.0%
C2DE	4594	43.0%
Geographic Region		
North	2553	23.9%
Midlands	1763	16.5%
East	963	9.0%
London	1442	13.5%
South	2509	23.5%
England (net)	9231	86.4%
Wales	524	4.9%
Scotland	930	8.7%
Location		
Urban	8402	78.6%
Town and Fringe	1038	9.7%
Rural	1240	11.6%
Uncoded	3	0.0%
Working Status		
Working full time	4315	40.4%
Working part time	1579	14.8%
All workers (net)	5893	55.2%
Full time student	631	5.9%
Retired	2554	23.9%
Unemployed	586	5.5%
Not working/ Other	1020	9.5%
Marital Status		
Married/ Civil Partnership	4814	45.30%
Living as married	1447	13.60%
Separated/ Divorced	875	8.20%
Widowed	350	3.30%
Never Married	3144	29.60%
Children in Household		
0	7747	72.5%
1	1268	11.9%
2	1037	9.7%
3+	389	3.6%
Refused	243	2.3%
Social Media/Messaging Service		
Facebook	7508	76.5%
Twitter	3743	38.1%
LinkedIn	2034	20.7%
Pinterest	1484	15.1%
Instagram	4088	41.7%
Snapchat	1518	15.5%
Facebook Messenger	6625	67.5%
WhatsApp	7446	75.9%
Skype	1160	11.8%
Household Income		
Under £5,000 per year	222	2.2%
£5,000 to £9,999 per year	397	3.9%
£10,000 to £14,999 per year	641	6.2%
£15,000 to £19,999 per year	691	6.7%
£20,000 to £24,999 per year	829	8.0%
£25,000 to £29,999 per year	722	7.0%
£30,000 to £34,999 per year	674	6.5%
£35,000 to £39,999 per year	532	5.2%
£40,000 to £44,999 per year	568	5.5%
£45,000 to £49,999 per year	436	4.2%
£50,000 to £59,999 per year	635	6.2%
£60,000 to £69,999 per year	447	4.3%
£70,000 to £99,999 per year	702	6.8%
£100,000 to £149,999 per year	258	2.5%
£150,000 and over	100	1.0%
Don’t know	664	6.4%
Prefer not to answer	1781	17.3%
Personal Income		
Under £5,000 per year	764	7.5%
£5,000 to £9,999 per year	826	8.1%
£10,000 to £14,999 per year	1108	10.8%
£15,000 to £19,999 per year	1059	10.4%
£20,000 to £24,999 per year	1079	10.6%
£25,000 to £29,999 per year	783	7.7%
£30,000 to £34,999 per year	668	6.5%
£35,000 to £39,999 per year	437	4.3%
£40,000 to £44,999 per year	351	3.4%
£45,000 to £49,999 per year	239	2.3%
£50,000 to £59,999 per year	256	2.5%
£60,000 to £69,999 per year	149	1.5%
£70,000 to £99,999 per year	181	1.8%
£100,000 and over	94	0.9%
Don’t know	403	3.9%
Prefer not to answer	1821	17.8%

### Questionnaire results

Of the respondents, 5,494 (51.4%) correctly believed that medical cannabis is legal in the UK. Subsequently, 5,191 (48.6%) participants either did not know medical cannabis is legal (*n* = 3,310; 31.0%) or believed that it was illegal (*n* = 1,881; 17.6%). A small proportion of participants were already consuming CBD as a wellness product (*n* = 684; 6.4%), on prescription (*n* = 286; 2.7%), or for another reason (*n* = 222; 2.1%). For those who did not consume CBD products (*n* = 9,458), 2,933 (31.0%) respondents said they would like access to CBD for medical purposes if it were available to them at a cost that was reasonable to them. Most respondents (*n* = 10,076; 94.3%) were unaware of the requirements of over the counter CBD products to conform to novel foods regulations from April 2021 before taking the survey. Of those currently taking CBD wellness products most will either continue to source a product that conforms to novel foods regulations (*n* = 273; 39.9%) or consider inquiring as to whether they can receive CBD on prescription (*n* = 119; 17.4%). Most respondents were unaware as to whether there is a cost difference between over the counter CBD or CBD obtained via prescription (*n* = 7,261; 68.0%). On being asked, which is the main barrier to people discussing medical cannabis with a doctor in the UK, the most frequent responses were people: associating it with recreational cannabis (*n* = 2,686; 25.1%), being unsure if it was legal (*n* = 2276; 21.3%), being unsure as to what it is a treatment for (*n* = 1,863; 17.4%), and not knowing about it in general (*n* = 1,210; 11.3%). Only 293 (2.7%) cited cost as a barrier to discussing medical cannabis with a doctor. Full questionnaire results are detailed in Table 2.

**Table 2:** Questionnaire results

Questionnaires Questions	n	%
*Do you think that medical cannabis is or is not legal in the UK?*		
Medical cannabis is legal	5494	51.4%
Medical cannabis is not legal	3310	31.0%
Don’t know	1881	17.6%
*“CBD” is a chemical substance found in cannabis that is thought to have medical benefits. It will not get you high, because it does not contain the chemical in cannabis that makes you high. Some CBD products can currently be purchased without prescription. Do you personally use ANY CBD (Cannabidiol) products (e.g. oil, gummies, capsules etc.)? (Please select all that apply)*		
Yes - mostly for wellness purposes	684	6.4%
Yes - prescribed for a medical condition	286	2.7%
Yes - for another reason	222	2.1%
No	9459	88.5%
Prefer not to say	139	1.3%
*As a reminder, “CBD” is a chemical substance found in cannabis that is thought to have medical benefits. It will not get you high, because it does not contain the chemical in cannabis that makes you high. Some CBD products can currently be purchased without prescription. You previously said that you do not use any CBD products (e.g. oil, gummies, capsules etc.)…Would you personally like to have access to CBD (Cannabidiol) products for medical purposes, if it was available to you at a price you consider reasonable?*		
Yes, I would	2933	31.0%
No, I wouldn’t	3530	37.3%
Don’t know	2957	31.3%
Prefer not to say	38	0.4%
*After April 2021, the majority of current CBD (Cannabidiol) over the counter wellness products available will require Novel Foods validation in order to be sold to the general public. Before taking this survey, were you aware of this change?*		
Yes, I was	608	5.7%
No, I wasn’t	10,076	94.3%
*As a reminder, after April 2021, the majority of current CBD (Cannabidiol) over the counter wellness products available will require Novel Foods validation in order to be sold to the general public. You previously said that you do use CBD products for wellness purposes…With this in mind, which ONE, if any, of the following BEST applies to you? (Please select the option that best applies)*		
I will source another wellness product that contains CBD but can be legally purchased/ sold	273	39.9%
I will consider medical CBD (i.e. prescription required via my doctor/ medical practitioner)	119	17.4%
I will stop taking CBD completely	70	10.3%
None of these	75	10.9%
Don’t know	117	17.1%
Prefer not to say	30	4.4%
*For the following question, even if you do not use CBD (Cannabidiol) products, we are still interested in your opinion. Would you say that over the counter CBD products are more or less expensive than medical cannabis grade CBD via prescription charge in the UK, or would you say these cost about the same?*		
Over the counter CBD is more expensive	1635	15.3%
About the same	951	8.9%
Over the counter CBD is less expensive	837	7.8%
Don’t know	7261	68.0%
*As a reminder, even if you do not use CBD (Cannabidiol), we are still interested in your opinion. Which ONE, if any, of the following do you think is the MAIN barrier stopping people in the UK from using or speaking to their doctor about medical cannabis? (Please select the option that best applies)*		
It is too expensive	292	2.7%
People are not sure whether they are eligible or what ailments/ illnesses it can be used for	1863	17.4%
People do not know about it	1210	11.3%
People are unsure whether it is legal	2276	21.3%
People associate it with the recreational use of cannabis	2686	25.1%
Other	109	1.0%
Don’t know	1381	12.9%
Not applicable - I do not think there are any barriers in particular stopping people in the UK from using or talking to their doctor about medical cannabis	867	8.1%

Full results of the responses to each question analysed according to underlying demographics are detailed in supplementary data file 1.

### Legality of medical cannabis

Male respondents were more likely to know that medical cannabis is legal (54.3%) compared to female respondents (48.7%; *p* < 0.001). Participants in the age category 18–24 were less likely to know that medical cannabis is legal (47.6%) compared to those aged 45–54 (53.9; *p* = 0.003) and 55 years and older (54.8%; *p* < 0.001) respectively.

### Personal use of CBD

Women were more likely to consume CBD for wellness purposes (7.2%) compared to men (5.6%; *p* = 0.001). However, men were more likely to consume CBD when prescribed for a medical condition (3.3% vs. 2.1%; *p* < 0.001). Participants aged 35–44 (*p* < 0.005) 45–54 (*p* < 0.001) and 55 years and older (*p* < 0.001) were more likely to consume CBD for wellness and via a prescription compared to 18- to 24-year-olds. Participants from a working class background were more likely to receive CBD via prescription (3.3%) compared to those from upper and middle class backgrounds (2.2%; *p* = 0.002).

### Desire for access in the future for medical purposes

Men were also more likely to desire access to CBD for medicinal purposes in the future (32.9%) compared to women (29.2%; *p* < 0.001). Those aged 55 and over were less likely to desire access to CBD in the future compared to those aged between 18 and 24 (25.5% vs. 35.0%; *p* < 0.001).

### Awareness of the novel food regulatory status of CBD wellness products

Male respondents were more likely to be aware of the change in regulations surrounding the novel food status of CBD (6.4% vs. 5.0%; *p* = 0.002). Adults aged under 25 were similarly more likely to be aware of the change in regulations requiring CBD to meet novel food guidance compared to those aged 35 and older (*p* < 0.01).

### Knowledge of cost difference between medical cannabis grade CBD and CBD wellness products

The majority of both male and female respondents didn’t know whether over the counter CBD products were more or less expensive than medical cannabis grade CBD (67.5% vs. 68.4%; *p* = 0.296). Those aged under 25 were most likely to identify that medical cannabis is more expensive than CBD wellness products, however this was still only 10.3% of respondents in that category (*p* < 0.05).

### Barriers to medical cannabis in the UK

Male participants were more likely to think there are no barriers to accessing medical cannabis (9.8% vs. 6.5% *p* < 0.001). Women were more likely to cite the following as barriers to access compared to men: unsure what it could be used for, unsure of its legal status and its association with recreational use (*p* < 0.05). People from working class backgrounds classification were more likely to cite cost as a barrier to access (3.5% vs. 2.1%; *p* < 0.001).

## Discussion

This survey study, which utilised quota sampling and sample matching to the demographic profile of the UK population, has demonstrated that despite medical cannabis being legalised in November 2018, 48.6% of respondents are unaware of this change. Men and older individuals were more likely to be aware that it is legal. Most participants (94.3%) were also unaware of the change in regulations for over the counter CBD wellness products. A not insignificant proportion of respondents, however, were already consuming CBD for medical, wellness or other purposes (11.2%). The most frequently reported main barrier to medical cannabis was its association with recreational cannabis consumption (25.1%).

Whilst the findings of this study that 48.6% of respondents are unaware that medical cannabis is legal in the UK is surprising, this is supported by a survey of 1022 UK-based adults commissioned by Hill Dickinson in April 2020. This found that 46% of respondents were similarly unaware that medical cannabis is legal if prescribed by a doctor (Hill 2020). Whilst the lack of knowledge of the legality of medical cannabis may be attributable to the rapid process in which the law change came about (Monaghan et al. 2021), the failure for this to improve over 12 months from the Hill Dickinson survey suggests that this lack of awareness is multifactorial. In addition to the speed of legislative change, it is likely that its complexity also bars access for some sections of society (Tallon 2020). The alterations to scheduling in the Misuse of Drugs Regulations 2018, stipulated several conditions by which a product could be defined as a CBMP (Crime, Policing and Fire Group (CPFG) UK Home Office 2018). Moreover, this definition excluded synthetic cannabinoids. In June 2020, Epidyolex, a CBD isolate which has been licensed as an adjunctive treatment for drug resistant epilepsy in the setting of Dravet and Lennox-Gastaut syndromes, was moved to Schedule 5 exempting it from the stringent regulation surrounding other CBMPs (UK Home Office 2020). This complexity may therefore be a barrier to understanding, particularly in those with lower educational attainment. In the present study those who were employed were more likely to know that medical cannabis is legal (*p*< 0.050), however there was no formal assessment of educational attainment. The lack of physician education has been consistently highlighted as a barrier to prescribing (Dowden 2021; Case 2020; Alexander 2020), however a greater emphasis on making patients and the public aware of the legislation surrounding medical cannabis will be equally important in determining appropriate access. It is important to recognise that although these represent barriers to access, the National Institute for Health and Care Excellence guidance on CBMPs concluded that there is insufficient evidence to recommend the prescribing of unlicenced products (Case 2020). Therefore, one must consider how important it is to address public knowledge of medical cannabis in absence of this evidence.

Despite attempts to match respondents to the wider UK population. There are differences between the UK population and those who access medical cannabis which may be responsible for some of the demographic differences found. Male patients were much more likely to know that medical cannabis is legal (*p* < 0.001) and want access to CBD for medical reasons in the future (*p*< 0.001). Reports from the UK Medical Cannabis Registry have found that over 55% of patients are male, which may contribute to this finding (Ergisi et al. 2022). Moreover, the mean age of patients was 45 (Ergisi et al. 2022), which may also explain why those aged 45 or older were more likely to know about the legal status of CBMPs in the present study. However, further qualitative analysis would be required to understand the reasons why these disparities exist.

The respondents identified the association of medical cannabis with recreational consumption (25.1%), lack of knowledge of its legality (21.3%), and a paucity of information about what it could be used for (17.4%) as main barriers to accessing or even speaking to their doctor about medical cannabis. Stigma has been consistently identified as a barrier to prescribing, both within society and the medical profession in particular (Lashley & Pollock 2020; Schlag 2020; Troup et al. 2022). The history and driving forces of this stigma are complicated and largely date back to legislative change in the United States in the 1930s which has been co-opted globally, particularly by other Western nations (Lashley & Pollock 2020). Lashley & Pollock have previously described the necessary phases required to reduce medical cannabis stigma that rely upon initiating a moral agenda whereby medical cannabis is associated to positive values (Lashley & Pollock 2020). An example of this can already be seen in the UK, whereby the prescribing of medical cannabis in children with epilepsy has enhanced its identity as a medication (Monaghan et al. 2021). Further patient testimonies in other conditions where medical cannabis is more commonly prescribed, such as chronic pain, will help further reduce stigmatisation (Lashley & Pollock 2020). It is essential, however, that efforts to destigmatise and educate the population as to the medical uses and legality of medical cannabis are evidence-based and balanced to avoid over-stating its role within treatment pathways.

With regards to over the counter CBD wellness products, the majority of respondents (94.3%) were not aware as to the regulations which govern their safety. This is despite 6.4% already consuming CBD for wellness reasons and a further 2.1% for other reasons. In depth studies of public attitudes to complementary and alternative therapies, as well as dietary supplements has highlighted that communication of the risks and benefits of products is often unclear and utilise confusing jargon (Egan et al. 2011). Moreover, as they are not subject to the same restrictions in marketing as medical cannabis in the UK unsubstantiated claims can often be propagated through social media and other platforms (Merten et al. 2020). The findings of the present study reinforce the need for clear education of the differences between CBD wellness products and medical cannabis, in particular the differences in regulation.

Whilst the YouGov® polling method provides substantial benefits in assessing attitudes and beliefs by utilising quota sampling and post-hoc weighting that attempts to ensure the respondents proportionally match the demographics of the UK population, this does not mean that the study is without limitations. However, any sampling method cannot be wholly representative. YouGov® administers it surveys online and therefore may fail to capture the responses of those who either lack access to the internet or otherwise are less engaged online. The survey data is statistically weighted to the demographics of all UK adults, including those who lack internet access to attempt to control for this. However, there are inherent characteristics of people who do not have internet access that cannot be adjusted for despite these efforts. Whilst this study design helps to provide population level responses it does not allow for more in-depth qualitative analysis of public attitudes and beliefs. Ideally, this would subsequently be evaluated in a focus group or semi-structured interview setting in the future to gain greater depth to the understanding on public opinion on medical cannabis and CBD wellness products. Finally, data provided by YouGov® may only be analysed within Crunch.IO without access to the raw data which is prohibitive in presenting additional information from *Chi-squared* tests, such as standardised residuals, beyond that described in the results. Furthermore, multivariate statistical analysis also could not be performed due to limited access to the raw data for analysis using alternative statistical software.

## Conclusion

Despite almost two and a half years elapsing since legislative change in the UK made medical cannabis legal via prescription, a significant proportion of the population are still unaware of this change. Moreover, this lack of understanding is proposed by the public themselves as a main barrier to accessing medical cannabis, alongside a lack of education on how it might be used in a medical context and its association with recreational consumption. Similarly, the public are unaware of the regulations which over the counter CBD wellness products need to conform to, leaving them at risk of using the products inappropriately. These findings provide a clear directive to provide an educational offering to improve the awareness of the differing purposes of medical cannabis and wellness CBD products. Importantly, this must be evidence-based to ensure the risks and efficacy of both products are clearly communicated. This is likely to have a multifactorial effect of reducing stigma through expanding the moral agenda for medical cannabis in the UK beyond childhood epilepsy, whilst also ensuring that people who choose to access either medical cannabis or wellness CBD products do so safely according to the correct indication.

## Supplementary Information

**Additional file 1: Supplementary Table 1.** Questions and response options. **Supplementary Table 2.** Responses to question ‘Do you think that medical cannabis is or is not legal in the UK?’ stratified by demographics. **Supplementary Table 3.** Responses to question ‘“CBD” is a chemical substance found in cannabis that is thought to have medical benefits. It will not get you high, because it does not contain the chemical in cannabis that makes you high. Some CBD products can currently be purchased without prescription. Do you personally use ANY CBD (Cannabidiol) products (e.g. oil, gummies, capsules etc.)? (Please select all that apply)’ stratified by demographics. **Supplementary Table 4.** Responses to question ‘As a reminder, “CBD” is a chemical substance found in cannabis that is thought to have medical benefits. It will not get you high, because it does not contain the chemical in cannabis that makes you high. Some CBD products can currently be purchased without prescription. You previously said that you do not use any CBD products (e.g. oil, gummies, capsules etc.)... Would you personally like to have access to CBD (Cannabidiol) products for medical purposes, if it was available to you at a price you consider reasonable?’ stratified by demographics. **Supplementary Table 5.** Responses to question ‘After April 2021, the majority of current CBD (Cannabidiol) over the counter wellness products available will require Novel Foods validation in order to be sold to the general public. Before taking this survey, were you aware of this change?’ stratified by demographics. **Supplementary Table 6.** Responses to ‘For the following question, even if you do not use CBD (Cannabidiol) products, we are still interested in your opinion. Would you say that over the counter CBD products are more or less expensive than medical cannabis grade CBD via prescription charge in the UK, or would you say these cost about the same?’ according to demographics.

## Data Availability

All raw data contained within supplementary materials.
