# Supplementary material for: Medical cannabis, CBD wellness products and public awareness of evolving regulations in the United Kingdom
Source: J Cannabis Res. 2022 Oct 29;4:56. doi: 10.1186/s42238-022-00165-6 (PMC9617440; doi:10.1186/s42238-022-00165-6)
Supplement: Supplementary file 1 — Additional file 1: Supplementary Table 1. Questions and response options. Supplementary Table 2. Responses to question ‘Do you think that medical cannabis is or is not legal in the UK?’ stratified by demographics. Supplementary Table 3. Responses to question ‘“CBD” is a chemical substance found in cannabis that is thought to have medical benefits. It will not get you high, because it does not contain the chemical in cannabis that makes you high. Some CBD products can currently be purchased without prescription. Do you personally use ANY CBD (Cannabidiol) products (e.g. oil, gummies, capsules etc.)? (Please select all that apply)’ stratified by demographics. Supplementary Table 4. Responses to question ‘As a reminder, “CBD” is a chemical substance found in cannabis that is thought to have medical benefits. It will not get you high, because it does not contain the chemical in cannabis that makes you high. Some CBD products can currently be purchased without prescription. You previously said that you do not use any CBD products (e.g. oil, gummies, capsules etc.)... Would you personally like to have access to CBD (Cannabidiol) products for medical purposes, if it was available to you at a price you consider reasonable?’ stratified by demographics. Supplementary Table 5. Responses to question ‘After April 2021, the majority of current CBD (Cannabidiol) over the counter wellness products available will require Novel Foods validation in order to be sold to the general public. Before taking this survey, were you aware of this change?’ stratified by demographics. Supplementary Table 6. Responses to ‘For the following question, even if you do not use CBD (Cannabidiol) products, we are still interested in your opinion. Would you say that over the counter CBD products are more or less expensive than medical cannabis grade CBD via prescription charge in the UK, or would you say these cost about the same?’ according to demographics. [file 42238_2022_165_MOESM1_ESM.zip › Supplementary table 1.docx]

**Table 1.** Questions and response options

| **Topic** | **Required Logic** | **Question Text** | **Responses** |
| --- | --- | --- | --- |
| 1. Legality of Medical Cannabis | - | Do you think that medical cannabis is or is not legal in the UK? | - Medical cannabis is legal - Medical cannabis is not legal - Don't know |
| 2. Personal use of CBD | - | Cannabidiol (CBD) is a chemical substance found in cannabis that is thought to have medical benefits. It will not get you high, because it does not contain the chemical in cannabis that makes you high. Some CBD products can currently be purchased without prescription. Do you personally use any CBD products (e.g. oil, gummies, capsules etc.)? (Please select all that apply) | - Yes - mostly for wellness purposes - Yes - prescribed for a medical condition - Yes - for another reason - No - Prefer not to say |
| 2a. Desire for access in the future for medical purposes | Response to 2 – ‘No’ | As a reminder, Cannabidiol (CBD) is a chemical substance found in cannabis that is thought to have medical benefits. It will not get you high, because it does not contain the chemical in cannabis that makes you high. Some CBD products can currently be purchased without prescription. You previously said that you do not use any CBD products (e.g. oil, gummies, capsules etc.). Would you personally like to have access to CBD products for medical purposes, if it was available to you at a price you consider reasonable? | - Yes, I would - No, I wouldn't - Don't know - Prefer not to say |
| 3. Awareness of the novel foods status of CBD extracts | - | After April 2021, the majority of current Cannabidiol (CBD) over the counter wellness products available will require Novel Foods validation in order to be sold to the general public. Before taking this survey, were you aware of this change? | - Yes, I was - No, I wasn't |
| 3a. Desire to continue using CBD wellness products after novel foods status change | Response to 2 – ‘Yes - mostly for wellness purposes’ | As a reminder, after April 2021, the majority of current Cannabidiol (CBD) over the counter wellness products available will require Novel Foods validation in order to be sold to the general public. You previously said that you do use CBD products for wellness purposes. With this in mind, which ONE, if any, of the following BEST applies to you? (Please select the option that best applies) | - I will source another wellness product that contains CBD but can be legally purchased/ sold - I will consider medical CBD (i.e. prescription required via my doctor/ medical practitioner) - I will stop taking CBD completely - None of these - Don't know - Prefer not to say |
| 4. Knowledge of cost difference between medical cannabis grade CBD and CBD wellness products | - | For the following question, even if you do not use Cannabidiol (CBD) products, we are still interested in your opinion. Would you say that over the counter CBD products are more or less expensive than medical cannabis grade CBD via prescription charge in the UK, or would you say these cost about the same? | - Over the counter CBD is more expensive - About the same - Over the counter CBD is less expensive - Don’t know |
| 5. Barriers to medical cannabis in the UK | - | As a reminder, even if you do not use Cannabidiol (CBD), we are still interested in your opinion. Which ONE, if any, of the following do you think is the MAIN barrier stopping people in the UK from using or speaking to their doctor about medical cannabis? (Please select the option that best applies) | - It is too expensive - People are not sure whether they are eligible or what ailments/illnesses it can be used for - People do not know about it - People are unsure whether it is legal - People associate it with the recreational use of cannabis - Other - Don’t know - Not applicable - I do not think there are any barriers in particular stopping people in the UK from using or talking to their doctor about medical cannabis |
